# Superior diagnostic performance of Grocott methenamine silver staining in pulmonary cryptococcosis: a multicenter, large-sample cohort study

**DOI:** 10.3389/fmicb.2025.1615057

**Published:** 2025-06-04

**Authors:** Suijing Wang, Jieyi Lai, Chengyou Zheng, Pengfei Yang, Zhengyi Zhou, Haibo Wu, Mayan Huang, Xinke Zhang, Yongbo Xiao, Jierong Chen, Chao Ma, Keming Chen, Liyan Lin, Huanyu Liu, Yubo Cai, Xiaolei Xue, Zizi Li, Jiewei Chen

**Affiliations:** ^1^State Key Laboratory of Oncology in South China, Guangdong Provincial Clinical Research Center for Cancer, Guangzhou, China; ^2^Department of Pathology, Sun Yat-sen University Cancer Center, Guangzhou, China; ^3^Department of Pathology, The Fifth Affiliated Hospital, Sun Yat-sen University, Zhuhai, China; ^4^Clinical Oncology School of Fujian Medical University, Fujian Cancer Hospital, Fuzhou, China; ^5^Department of Pathology, Peking University Shenzhen Hospital, Shenzhen, China; ^6^Department of Pathology, Jiangmen Central Hospital, Jiangmen, China; ^7^Department of Pathology, Nanfang Hospital, Southern Medical University, Guangzhou, China

**Keywords:** GMS, PAS, AB, histology, pulmonary cryptococcosis

## Abstract

**Introduction:**

In the histopathological diagnosis of pulmonary fungal infections, particularly pulmonary cryptococcosis, the diagnostic performance of different staining techniques varies significantly, often confounding pathologists. This study aims to systematically analyze Grocott methenamine silver (GMS), periodic acid-Schiff (PAS), and alcian blue (AB) staining methods to establish evidence-based diagnostic criteria.

**Methods:**

We incorporated histopathological data from 790 cases of pulmonary cryptococcosis that were definitively diagnosed in six tertiary hospitals. Multidimensional statistical analyses were performed to evaluate the performance of GMS, PAS, and AB staining methods.

**Results:**

GMS staining had a 100% positive diagnostic rate among all cohorts, which was significantly higher than that of PAS staining (93.7%, *p <* 0.001) and AB staining (75.4%, *p <* 0.001). Further statistical analyses indicated that GMS was superior to PAS and AB in detecting the number of cryptococci and in demonstrating the staining intensities for both intracellular and extracellular cryptococci (*p* < 0.001 for all comparisons). In the necrotic cores and peri-necrotic margins of granulomas, GMS more clearly localized cryptococci and detected a higher fungal burden. Even in colonies with minimal polysaccharides in fungal cell walls and capsules, GMS exhibited high sensitivity and provided clear visualization.

**Conclusion:**

GMS staining is the best method for diagnosing pulmonary cryptococcosis because of its high sensitivity and excellent visualization capabilities. Using GMS alone can meet the requirements for diagnostic accuracy, and we recommend GMS as the gold standard for histopathological confirmation of pulmonary cryptococcosis.

## Introduction

Pulmonary cryptococcosis (PC), a serious fungal lung infection caused by *Cryptococcus* species, has become a growing public health concern (Howard-Jones et al., 2022). Although over 30 species exist, *Cryptococcus neoformans* and *Cryptococcus gattii* constitute the primary pathogenic species. Notably, *Cryptococcus neoformans* is ranked highest in the fungal priority pathogens list recently released by the World Health Organization (Zhao et al., 2023; World Health Organization, 2023). Cryptococcosis is primarily acquired via respiratory aspiration (Chen et al., 2022). Following pulmonary invasion, alveolar macrophages play a pivotal role in innate immunity by recognizing, phagocytosing, and eliminating cryptococci through diverse mechanisms (Wang Y. et al., 2022). Multinucleated giant cells, formed by the macrophage fusion at infection sites, phagocytose and sequester pathogens to limit their dissemination into surrounding tissues. These immune responses frequently induce granulomatous lesions that typically manifest as nodules on radiological imaging in PC. Despite the widespread clinical implementation of high-resolution computed tomography, distinguishing PC from pulmonary malignancies and tuberculosis remains challenging due to overlapping radiological features (Qu et al., 2020; Xin et al., 2022). Consequently, a definitive diagnosis typically requires histopathological confirmation through percutaneous lung biopsy, bronchial biopsy, or open thoracic surgery.

However, the diagnostic performance of histopathological methods for pulmonary fungal diseases still lacks sufficient clinical evidence. Pathologists are often uncertain when selecting diagnostic strategies due to the lack of consensus on optimal approaches. There are significant differences in the choice of diagnostic methods among pathology centers, with most hospital pathology departments using combined staining approaches [e.g., Grocott methenamine silver [GMS] combined with periodic acid-Schiff (PAS) and/or alcian blue (AB)]. Nevertheless, these methods exhibit marked performance variability in clinical practice. Both PAS and AB stain tissue components with colors similar to fungal elements, a feature that interferes with cryptococcal identification and reduces diagnostic accuracy. Critically, pulmonary granulomatous lesions have diverse etiologies, including non-infectious granulomatosis, tuberculosis, and other fungal pathogens (Rosen, 2022). False-negative staining results may prompt clinicians to consider alternative diagnoses, which could prolong the diagnostic process and delay antifungal therapy.

Our previous study showed that GMS staining is the most sensitive method for detecting pulmonary *Cryptococcus* (Wang S. et al., 2022). To further elucidate the superiority and diagnostic sufficiency of GMS staining for PC, we conducted a multicenter study involving 790 confirmed cases from six tertiary hospitals. In this study, digital pathology was employed for the first time to detect *Cryptococcus* quantitatively. We multidimensionally compared the sensitivity and accuracy of GMS, PAS, and AB staining methods in diagnosing PC, providing evidence-based scientific support for precise clinicopathologic diagnostic criteria.

## Materials and methods

### Patients and special staining sections

In this multicenter retrospective cohort study, 790 patients diagnosed with PC were included from six tertiary hospitals in China between April 2007 and November 2023. All patients presented with pulmonary nodules detected on radiographic imaging, and cryptococcal infection was histopathologically confirmed using special staining methods, including GMS, PAS, and AB. Inclusion criteria were that lung tissue sections were positive for *Cryptococcus* by at least one of the above staining methods. Radiologic findings and special staining results were extracted from institutional electronic health records. All corresponding stained slides were collected for centralized pathological review. The study was approved by the Institutional Review Board of Sun Yat-sen University Cancer Center (B2021-452-Y03), and all procedures were conducted in accordance with international ethical guidelines for biomedical research involving human subjects.

### Special staining and evaluation

Special staining slides were prepared at each center by professionally trained histotechnologists following standardized staining protocols. All specimens were sectioned into 4–5 μm thick serial sections from formalin-fixed paraffin-embedded blocks.

GMS staining utilized 8% chromic acid as the oxidizing agent and methenamine-silver-nitrate solution as the reaction medium, followed by light green counterstaining. PAS staining involved periodic acid oxidation, incubation with Schiff’s reagent in darkness, and counterstaining with Mayer’s hematoxylin. AB staining employed alcian blue solution (pH 2.5) and counterstaining with nuclear fast red. Positive diagnostic criteria for GMS, PAS, and AB staining were defined as the appearance of *Cryptococcus* in brown-black, magenta, and blue, respectively.

GMS-, PAS-, and AB-stained sections from all PC cases were independently reviewed by two experienced pathologists. A random subset of 68 cases was selected for staining evaluation. Whole-slide images were scanned using an Aperio AT2 digital pathology scanner (Leica, Germany). The number of positive cryptococci under GMS, PAS, and AB staining was subsequently counted using QuPath v.0.5.1 software (Queen’s University, Belfast, Northern Ireland) (Bankhead et al., 2017). For microscopic evaluation, the staining intensities of intracellular cryptococci (IC) and extracellular cryptococci (EC) in non-necrotic areas were evaluated in three high-power fields per case. The staining intensities of cryptococci were evaluated in both necrotic cores and their peri-necrotic margins. Staining intensity scores ranged from 0 to 2 (0, negative; 1, weak; 2, strong). Two pathologists independently scored all cases, with discrepancies resolved by consensus.

### Statistical analysis

The correlations between two categorical variables were assessed using McNemar’s or McNemar-Bowker tests, as appropriate. Continuous variables were compared using paired t-tests for normally distributed data. Non-normally distributed data were compared using Wilcoxon signed-rank tests, combined with a bootstrapping procedure to test for differences in medians with 10,000 iterations. *p*-values less than 0.05 were considered statistically significant. All analyses were performed using SPSS v25.0 (IBM Corp., Armonk, NY, USA), GraphPad Prism 9 (GraphPad Software Inc., San Diego, CA, USA), and Python 3.13.3 (Python Software Foundation, Beaverton, OR, USA).

## Results

### Cohort characteristics across participating centers

**Figure 1:**
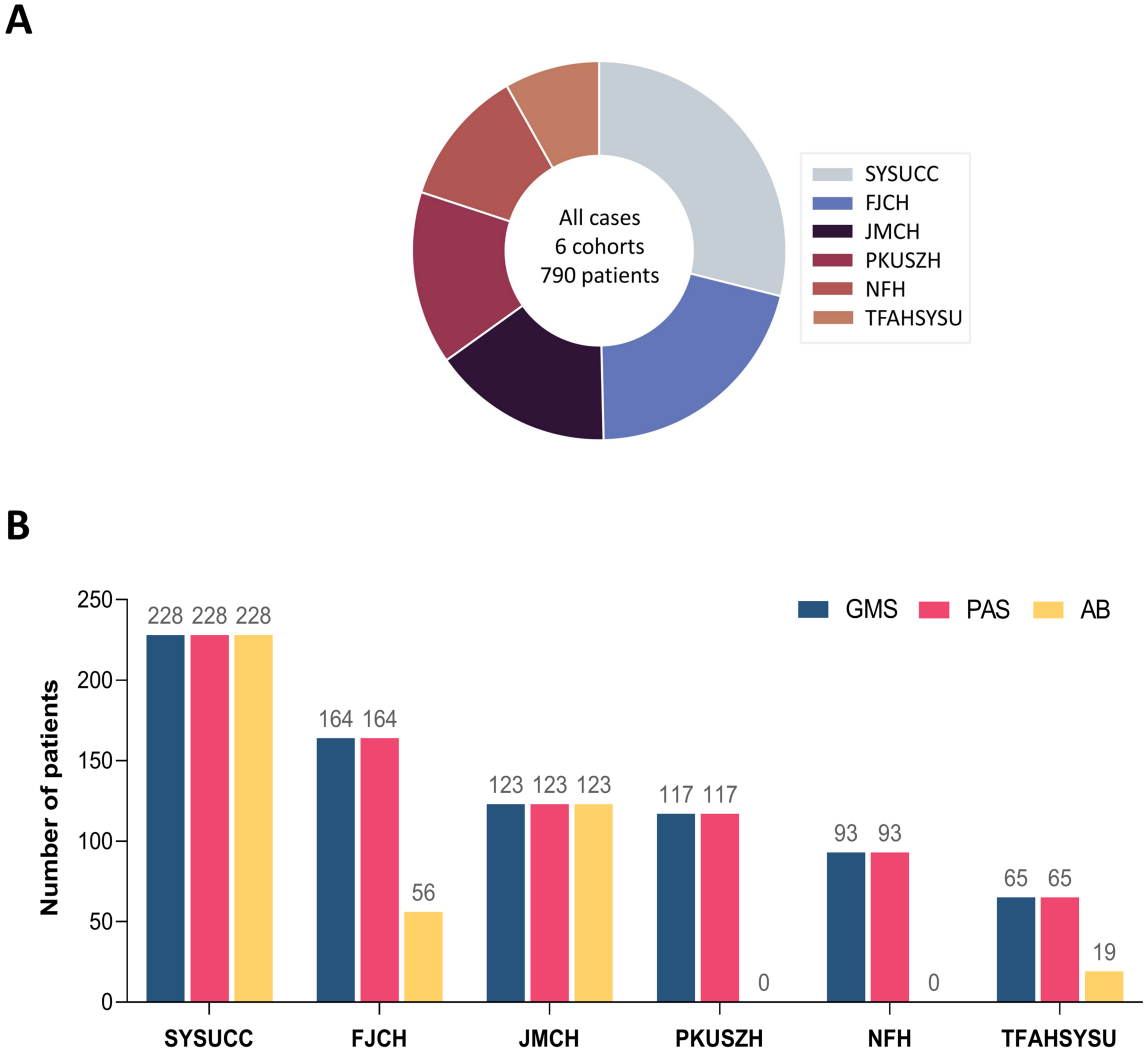
Overview of six multicenter cohorts with pulmonary cryptococcosis. **(A)** Patient distribution across six multicenter cohorts. **(B)** Distribution of staining methods across centers.

In this study, data from 790 patients with PC were retrospectively collected from six centers and then included in the analysis. Sun Yat-sen University Cancer Center (SYSUCC) contributed 28.9% (228/790) of the cases, followed by Fujian Cancer Hospital (FJCH, 20.8%, 164/790), Jiangmen Central Hospital (JMCH, 15.6%, 123/790), Peking University Shenzhen Hospital (PKUSZH, 14.8%, 117/790), Nanfang Hospital (NFH, 11.8%, 93/790), and The Fifth Affiliated Hospital of Sun Yat-sen University (TFAHSYSU, 8.2%, 65/790). The distribution is presented in Figure 1A.

All centers employed GMS and PAS staining methods for the diagnosis of PC. A subset of 426 patients (53.9%) from four centers (SYSUCC, JMCH, FJCH, and TFAHSYSU) underwent the GMS-PAS-AB combined staining approach. The distribution of staining methods across centers is detailed in Figure 1B.

### GMS positivity rates reached 100%, with diagnostic accuracy consistent between GMS alone and combined approaches

**Figure 2:**
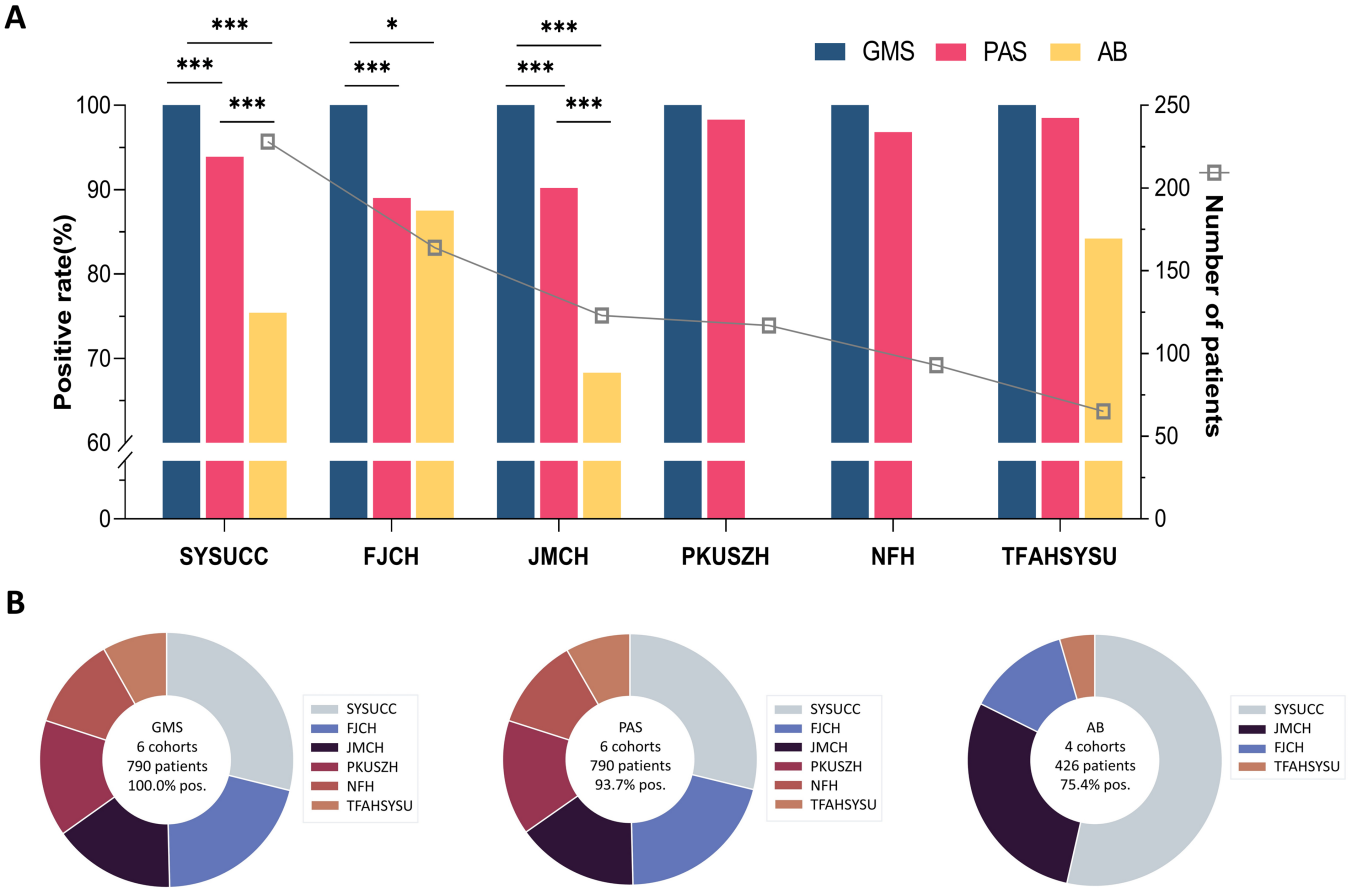
Diagnostic sensitivity comparison of staining methods in six pulmonary cryptococcosis cohorts. **(A)** Positive detection rates of GMS, PAS, and AB staining in each cohort. **(B)** Aggregate positivity rates and case distribution patterns. (* *p <* 0.05, *** *p <* 0.001).

The results of GMS, PAS, and AB staining for detecting cryptococci are summarized in Table 1. In the SYSUCC cohort, the positivity rates for GMS, PAS, and AB staining were 100.0, 93.9, and 75.4%, respectively. GMS positivity was significantly higher than those of PAS (*p* < 0.001) and AB (*p <* 0.001). In the FJCH cohort, the positivity rates for GMS, PAS, and AB staining were 100.0, 89.0, and 87.5%, respectively. GMS positivity was significantly higher than those of PAS (*p* < 0.001) and AB (*p* = 0.016). Similarly, in the JMCH cohort, GMS positivity remained at 100.0%, significantly higher than those of PAS (90.2%, *p* < 0.001) and AB (68.3%, *p* < 0.001). Across all centers, GMS staining achieved a 100.0% positivity rate, outperforming PAS staining (89.0–98.5%) and AB staining (68.3–87.5%) (Figure 2A). Among 790 cases, GMS staining showed an overall positivity rate of 100.0% (790/790), compared to 93.7% (740/790) for PAS staining and 75.4% (321/426) for AB staining (*p <* 0.001 for GMS vs. PAS and GMS vs. AB; Figure 2B).

**Table 1:** Diagnostic results of GMS, PAS, and AB staining for pulmonary cryptococcosis across six multicenter cohorts.

Cohort	Staining	Negative (*n*/%)	Positive (*n*/%)	Total (*n*)	*p* value^a^		
					GMS VS PAS	GMS VS AB	PAS VS AB
SYSUCC					< 0.001	< 0.001	< 0.001
	GMS	0 (0.0)	228 (100.0)	228			
	PAS	14 (6.1)	214 (93.9)	228			
	AB	56 (24.6)	172 (75.4)	228			
FJCH					< 0.001	0.016	1.0
	GMS	0 (0.0)	164 (100.0)	164			
	PAS	18 (11.0)	146 (89.0)	164			
	AB	7 (12.5)	49 (87.5)	56			
JMCH					< 0.001	< 0.001	< 0.001
	GMS	0 (0.0)	123 (100.0)	123			
	PAS	12 (9.8)	111 (90.2)	123			
	AB	39 (31.7)	84 (68.3)	123			
PKUSZH					0.5	−	−
	GMS	0 (0.0)	117 (100.0)	117			
	PAS	2 (1.7)	115 (98.3)	117			
NFH					0.25	−	−
	GMS	0 (0.0)	93 (100.0)	93			
	PAS	3 (3.2)	90 (96.8)	93			
TFAHSYSU					1.0	0.25	0.25
	GMS	0 (0.0)	65 (100.0)	65			
	PAS	1 (1.5)	64 (98.5)	65			
	AB	3 (15.8)	16 (84.2)	19			
Total					< 0.001	< 0.001	< 0.001
	GMS	0 (0.0)	790 (100.0)	790			
	PAS	50 (6.3)	740 (93.7)	790			
	AB	105 (24.6)	321 (75.4)	426			

a McNemar’s test; − Not applicable.

SYSUCC, Sun Yat-sen University Cancer Center; FJCH, Fujian Cancer Hospital; JMCH, Jiangmen Central Hospital; PKUSZH, Peking University Shenzhen Hospital; NFH, Nanfang Hospital; TFAHSYSU, The Fifth Affiliated Hospital of Sun Yat-sen University.

As shown in Table 2, GMS staining alone and its combinations with PAS (GMS-PAS) or both PAS and AB (GMS-PAS-AB) achieved 100.0% positivity rates. However, combining multiple methods did not enhance diagnostic accuracy compared to GMS alone, highlighting the consistent efficacy of both individual and combined approaches.

**Table 2:** Detection rates of combined staining approaches in six pulmonary cryptococcosis cohorts.

Staining panel	Cohort	Positive (*n*/%)			Combined positive (*n*/%)
		GMS	PAS	AB	
GMS, PAS	PKUSZH	117 (100.0)	115 (98.3)	−	117 (100.0)
	NFH	93 (100.0)	90 (96.8)	−	93 (100.0)
	FJCH	108 (100.0)	97 (89.8)	−	108 (100.0)
	TFAHSYSU	46 (100.0)	45 (97.8)	−	46 (100.0)
	Total	364 (100.0)	347 (95.3)	−	364 (100.0)
GMS, PAS, AB	SYSUCC	228 (100.0)	214 (93.9)	172 (75.4)	228 (100.0)
	JMCH	123 (100.0)	111 (90.2)	84 (68.3)	123 (100.0)
	FJCH	56 (100.0)	49 (87.5)	49 (87.5)	56 (100.0)
	TFAHSYSU	19 (100.0)	19 (100)	16 (84.2)	19 (100.0)
	Total	426 (100.0)	393 (92.3)	321 (75.4)	426 (100.0)

− Not applicable. SYSUCC, Sun Yat-sen University Cancer Center; FJCH, Fujian Cancer Hospital; JMCH, Jiangmen Central Hospital; PKUSZH, Peking University Shenzhen Hospital; NFH, Nanfang Hospital; TFAHSYSU, The Fifth Affiliated Hospital of Sun Yat-sen University.

### GMS staining detected significantly more cryptococci than PAS and AB staining

**Figure 3:**
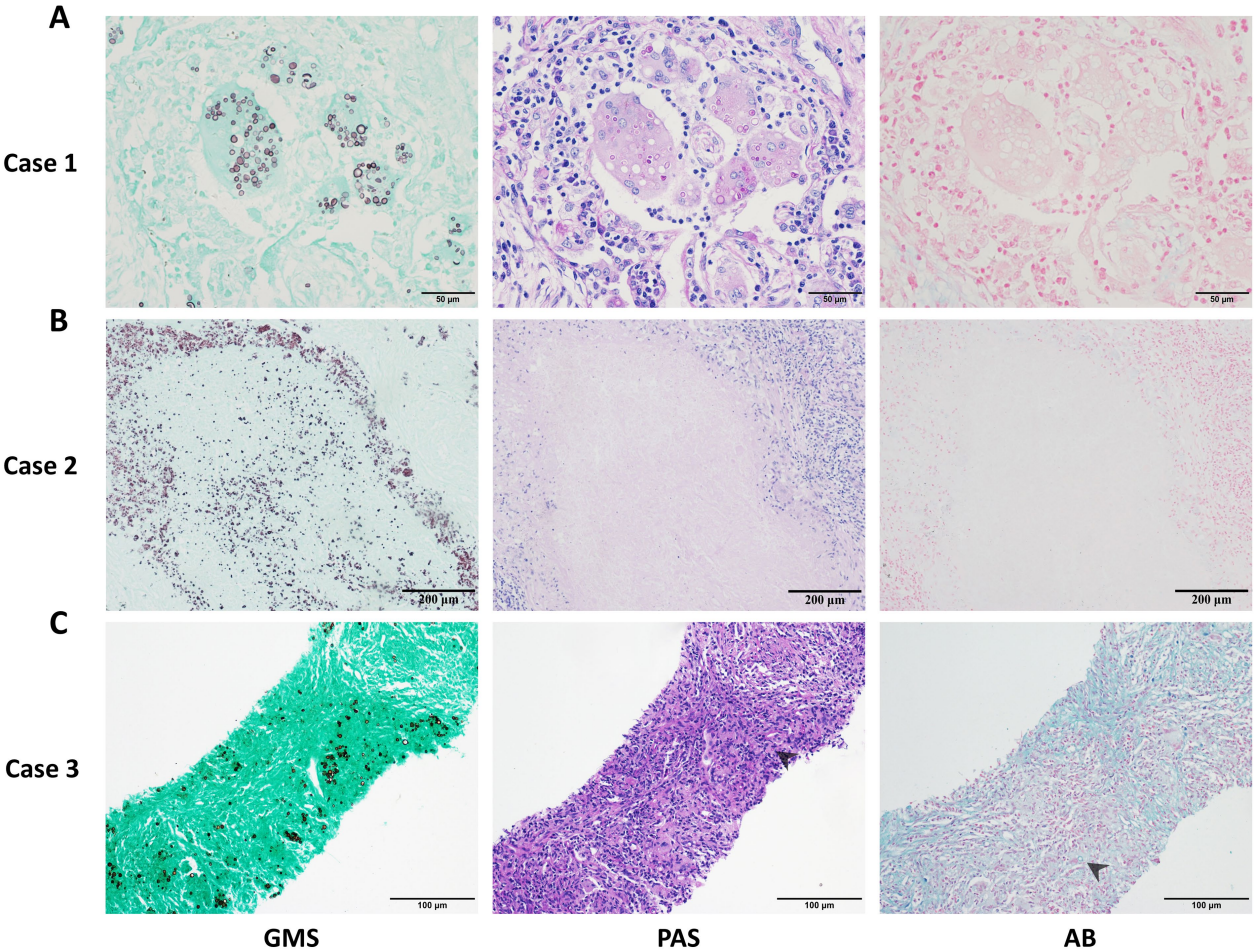
Representative cases of pulmonary cryptococcosis using GMS, PAS, and AB staining methods, respectively. **(A)** Case 1: Lung tissue with GMS+, PAS+, and AB− staining. **(B)** Case 2: Necrotic lung tissue with GMS+, PAS−, and AB− staining. **(C)** Case 3: Concurrent positivity in GMS, PAS, and AB staining with chromogenic interference (PAS and AB), compromising fungal discrimination (arrows indicate cryptococci).

Through microscopic analysis of cases from different centers, we observed that the GMS staining method had higher sensitivity and effectively identified more cryptococci. In contrast, PAS and AB were less sensitive than GMS and could even result in false-negative findings (Figures 3A,B). Additionally, PAS and AB stains produced substantial background noise and poor morphological clarity, which complicated the identification of cryptococci in samples with abundant mucus or collagen fibers (Figure 3C).

**Figure 4:**
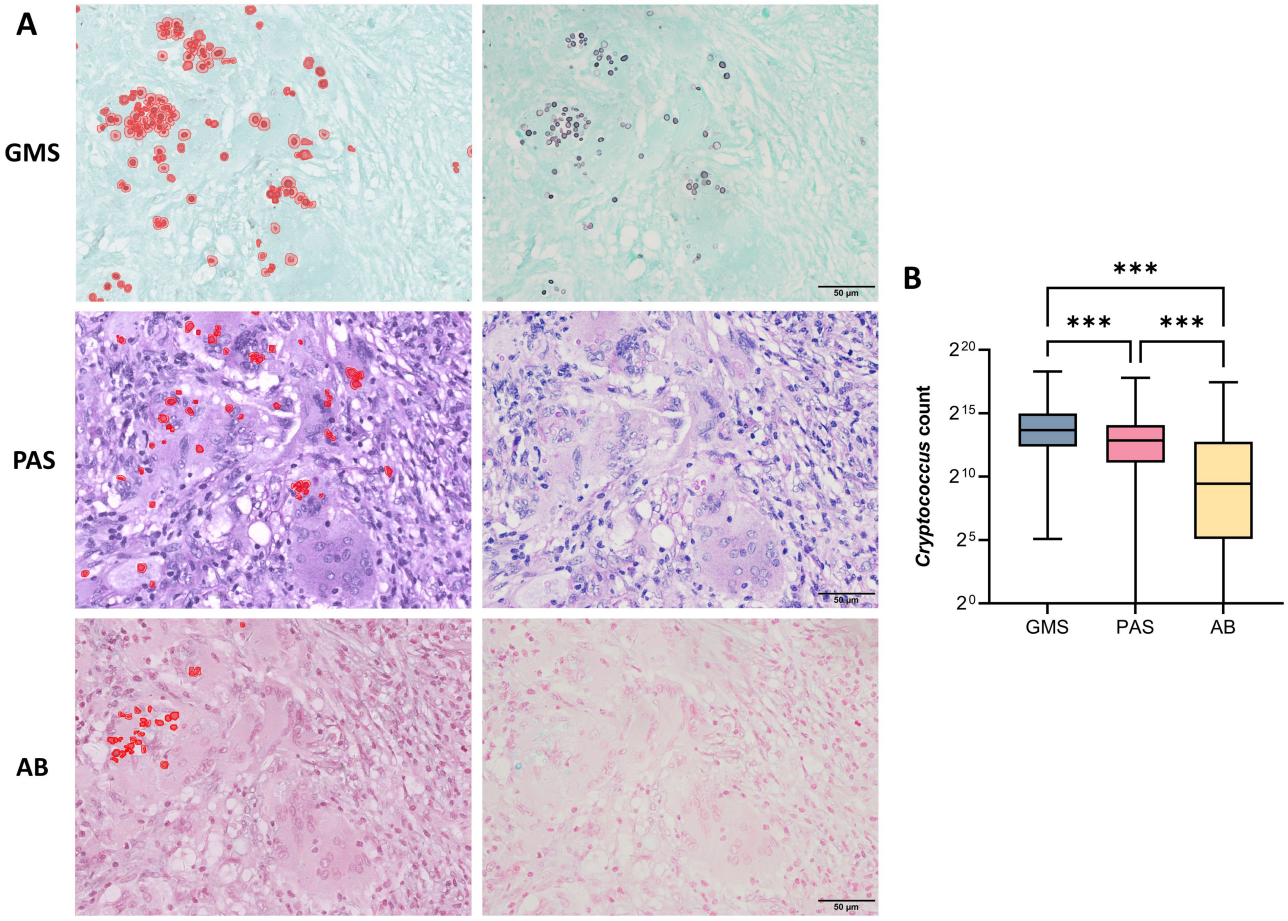
Detection efficiency of GMS, PAS, and AB staining for pulmonary cryptococci using digital tissue image analysis. **(A)** Left: Distribution pattern of positive cryptococcal counts identified by digital tissue image analysis. Right: Corresponding microscopic view of the same field. **(B)** Statistical comparison of positive cryptococcal counts detected by three staining methods. The Wilcoxon signed-rank test revealed that GMS had significantly greater detection efficacy than PAS and AB (*p* < 0.001 for both comparisons). (*** *p <* 0.001).

Figure 4A displays the distribution pattern of positive cryptococcal counts identified by digital tissue image analysis, alongside the corresponding microscopic image of the same field of view. As indicated in Supplementary Table S1, considerable differences were observed in the cryptococci detection counts among the three staining methods. GMS demonstrated superior performance, with the highest median (13,026) and mean values (31,329 ± 56,535), followed by PAS (median, 7,408; mean, 19,905 ± 40,908) and AB (median, 697; mean, 10,616 ± 29,162). The Wilcoxon signed-rank test revealed that GMS had significantly greater detection efficacy than PAS and AB (*p <* 0.001 for GMS vs. PAS and GMS vs. AB; Figure 4B). The median difference between GMS and PAS was 5,618 (95% CI: 3,275–8,428), and that between GMS and AB was 12,330 (95% CI: 8,746–16,564). Notably, GMS showed a detection range of 34 to 320,600 without zero values, achieving 100% accuracy. These findings indicate that GMS staining is significantly more sensitive for detecting cryptococci than PAS and AB staining.

### Correlation between GMS, PAS, and AB staining intensities and cryptococcal distribution locations

**Figure 5:**
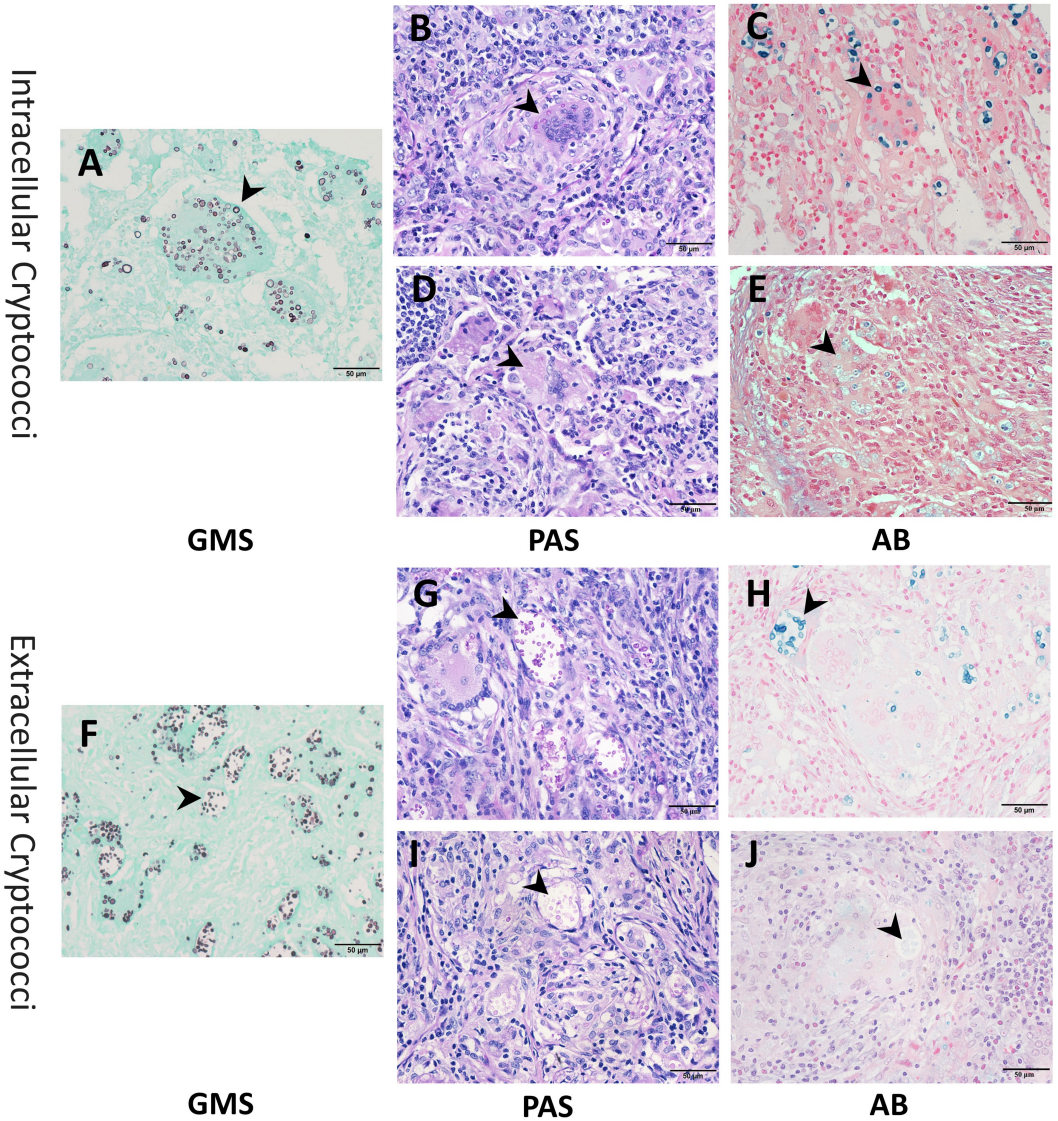
Representative images of intracellular and extracellular cryptococci using GMS, PAS, and AB staining. **(A–C)** Strong staining (arrow) of intracellular cryptococci across all methods. **(D,E)** Weak staining (arrow) of intracellular cryptococci with PAS and AB. **(F–H)** Strong staining (arrow) of extracellular cryptococci across all methods. **(I,J)** Weak staining (arrow) of extracellular cryptococci with PAS and AB.

**Figure 6:**
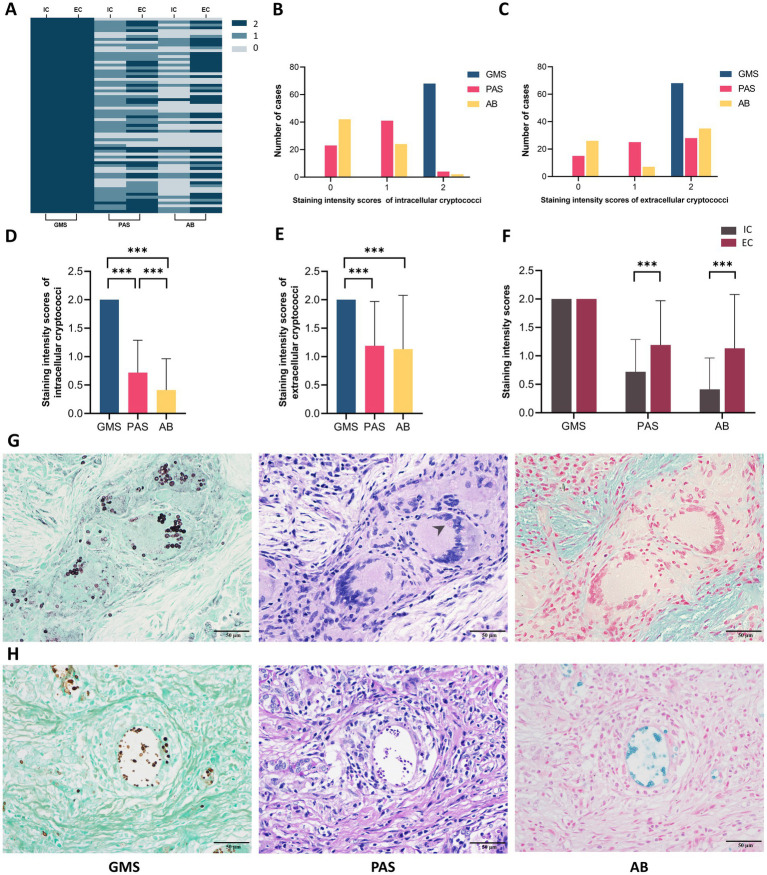
Staining intensities analysis of intracellular and extracellular cryptococci in 68 pulmonary cryptococcosis cases. **(A)** Heatmap of intensities scores by cryptococcal localization and staining method. **(B)** Case distribution across intensities scores for intracellular cryptococci. **(C)** Case distribution across intensities scores for extracellular cryptococci. **(D)** Correlation between staining methods and intracellular cryptococcal intensities. **(E)** Correlation between staining methods and extracellular cryptococcal intensities. **(F)** Comparative intensities scores between intracellular and extracellular cryptococci. **(G)** Representative cases of intracellular cryptococci that exhibit strong positive GMS, weak positive PAS (arrow), and negative AB. **(H)** Representative cases of extracellular cryptococci that exhibit strong positive GMS, PAS, and AB. IC, intracellular cryptococci; EC, extracellular cryptococci. (*** *p <* 0.001).

We further investigated the correlation between the staining intensities of three methods and the locations of cryptococcal distribution in 68 PC cases through microscopic analysis. The results showed that both IC and EC exhibited strong staining with GMS but variable staining intensities with PAS and AB (Figure 5). The strong positivity rate of GMS staining was significantly higher than those of PAS and AB staining for both IC (100.0% vs. 5.9% vs. 2.9%) and EC (100.0% vs. 41.2% vs. 51.5%), with *p* < 0.001 for all comparisons. The strong positivity rates of PAS (41.2% vs. 5.9%, *p <* 0.001) and AB staining (51.5% vs. 2.9%, *p <* 0.001) were significantly higher in EC than in IC (Table 3). Furthermore, we compared the intensity values of the three staining methods for IC and EC (Figures 6A–F). GMS staining exhibited higher intensities for both IC and EC compared to PAS and AB staining (*p* < 0.001 for all comparisons). EC also showed significantly greater staining intensities than IC in both PAS and AB methods (*p* < 0.001).

**Table 3:** Staining results for intracellular and extracellular cryptococci in 68 cases of pulmonary cryptococcosis using GMS, PAS, and AB staining.

Staining	Location	Negative (*n*/%)	Positive (*n*/%)		Total (*n*)	*p* value^a^
			Weak	Strong		
GMS						1.0
	Intracellular cryptococci	0 (0.0)	0 (0.0)	68 (100.0)	68	
	Extracellular cryptococci	0 (0.0)	0 (0.0)	68 (100.0)	68	
PAS						< 0.001
	Intracellular cryptococci	23 (33.8)	41 (60.3)	4 (5.9)	68	
	Extracellular cryptococci	15 (22.1)	25 (36.8)	28 (41.2)	68	
AB						< 0.001
	Intracellular cryptococci	42 (61.8)	24 (35.3)	2 (2.9)	68	
	Extracellular cryptococci	26 (38.2)	7 (10.3)	35 (51.5)	68	

a McNemar-Bowker test.

Microscopically, GMS staining revealed both IC and EC as dark brownish-black structures, which exhibited strong contrast against a bright green background. In contrast, PAS staining revealed fewer IC with weaker staining and poor contrast. AB staining even yielded false-negative results for IC detection. Compared to EC, IC displayed reduced staining intensity in both PAS and AB methods, suggesting that phagocytic uptake may lead to decreased histochemical reactivity (Figures 6G,H). Moreover, the number of cryptococci visualized by PAS and AB staining was significantly lower than that observed with GMS staining.

### GMS staining revealed higher cryptococci positivity and density in necrotic cores and peri-necrotic margins

**Figure 7:**
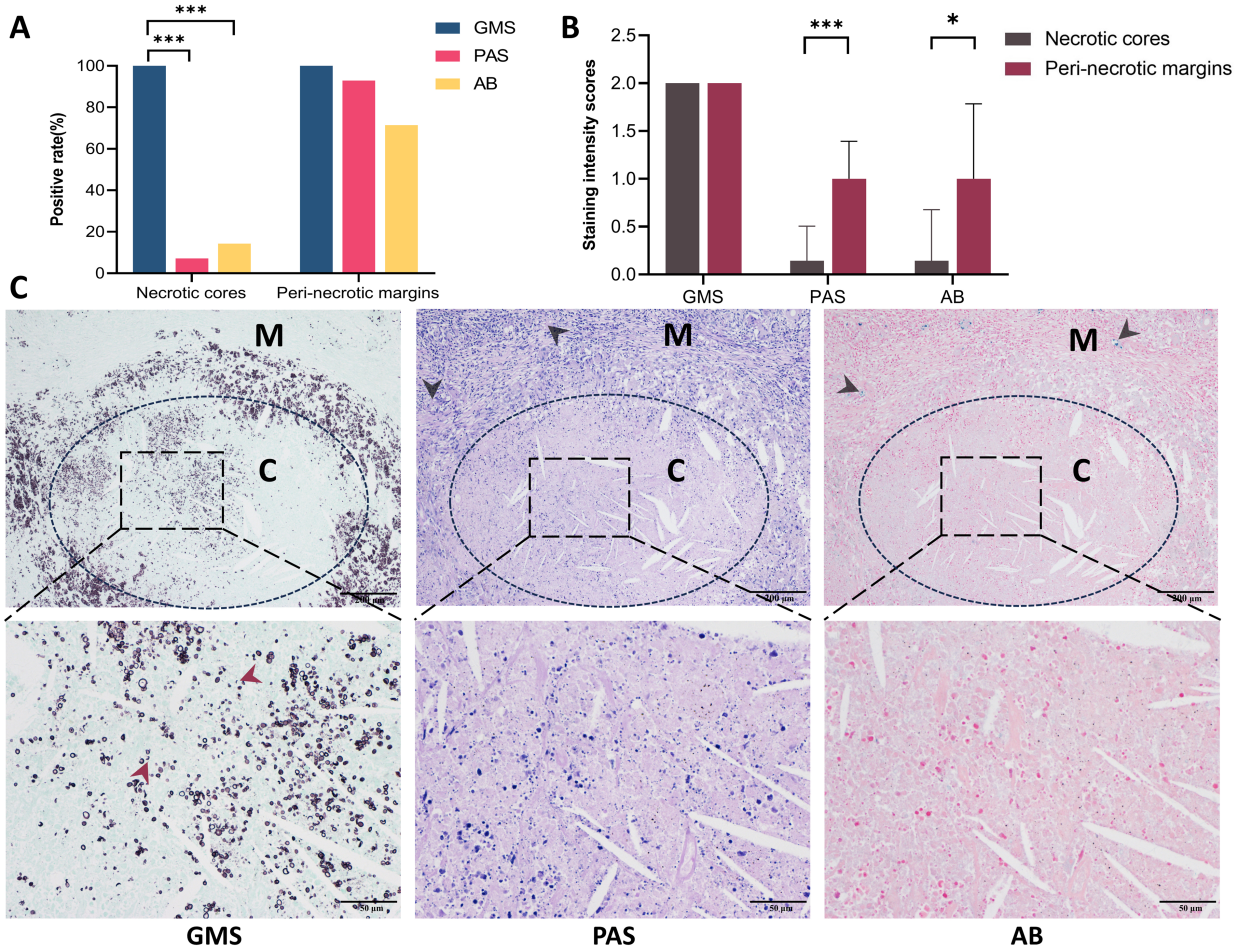
Comparison of GMS, PAS, and AB staining in necrotic cores versus peri-necrotic margins in pulmonary cryptococcosis. **(A)** Positive detection rates of three staining methods in necrotic cores and peri-necrotic margins. **(B)** Cryptococcal intensities scores in necrotic cores versus peri-necrotic margins across staining methods. **(C)** Representative images demonstrate that GMS provides superior fungal staining compared to PAS or AB, with cryptococci density in the peri-necrotic margins being significantly higher than in the necrotic cores (black arrows indicate the positive cryptococci in the peri-necrotic margins; red arrows indicate cryptococcal fragments under GMS staining). C, necrotic cores; M, peri-necrotic margins. (* *p <* 0.05, *** *p <* 0.001).

Histopathological analysis of PC specimens further identified necrotic foci in 20.6% (14/68) of cases. In the necrotic cores, GMS staining demonstrated complete positivity (14/14, 100%), which significantly outperformed PAS (1/14, 7.1%, *p <* 0.001) and AB (2/14, 14.3%, *p <* 0.001) staining (Figure 7A). In the peri-necrotic margins, the positivity rates for GMS, PAS, and AB were 100% (14/14), 92.9% (13/14), and 71.4% (10/14), respectively; in particular, the strong positivity rate of GMS was significantly higher than that of PAS (100.0% vs. 7.1%, *p* = 0.0015) and AB (100.0% vs. 28.6%, *p* = 0.0067) (Table 4). Additionally, the positivity rates and staining intensities of PAS and AB were significantly higher in the peri-necrotic margins than in the necrotic cores (*p* < 0.05 for all comparisons; Figure 7B).

**Table 4:** Staining results for cryptococci in necrotic cores and peri-necrotic margins using GMS, PAS, and AB staining.

Staining	Region	Negative (*n*/%)	Positive (*n*/%)		Total (*n*)	*p* value^a^
			Weak	Strong		
GMS						1.0
	Necrotic cores	0 (0.0)	0 (0.0)	14 (100.0)	14	
	Peri-necrotic margins	0 (0.0)	0 (0.0)	14 (100.0)	14	
PAS						0.003
	Necrotic cores	13 (92.9)	1 (7.1)	0 (0.0)	14	
	Peri-necrotic margins	1 (7.1)	12 (85.7)	1 (7.1)	14	
AB						0.046
	Necrotic cores	12 (85.7)	1 (7.1)	1 (7.1)	14	
	Peri-necrotic margins	4 (28.6)	6 (42.9)	4 (28.6)	14	

a McNemar-Bowker test.

Microscopically, the distribution density of cryptococci labeled by GMS was higher than that observed with PAS or AB staining in both necrotic cores and peri-necrotic margins of the same specimen. GMS staining revealed a higher density of cryptococci in the peri-necrotic margins compared to the necrotic cores. In the necrotic cores, GMS clearly demonstrated apoptotic or fragmented cryptococci, a feature not discernible with PAS or AB. Notably, sections stained with PAS and AB exhibited potential false-negative results (Figure 7C).

## Discussion

Special staining remains the gold standard for identifying fungal diseases in histopathology because it highlights key morphological features essential for pathogen identification. However, clinical practice lacks consensus regarding the selection of fungal diagnostic methods, with pathology departments adopting different staining techniques based on their institutional experience. To address this lack of standardization, we conducted a multicenter validation study analyzing 790 PC cases from six tertiary hospitals.

The statistical results demonstrated 100% sensitivity for GMS staining across all centers, outperforming PAS (89.0–98.5%) and AB (68.3–87.5%) staining. Overall, GMS achieved perfect diagnostic sensitivity (790/790), while PAS detected 93.7% (740/790) of cases, and AB detected 75.4% (321/426). Statistical analysis confirmed that GMS had significantly higher sensitivity than both PAS (*p* < 0.001) and AB (*p* < 0.001). The diagnostic accuracy of the GMS single method was already excellent and did not differ from that of combined staining approaches. Moreover, digital tissue image analysis in 68 representative cases revealed that GMS-stained sections had significantly higher cryptococcal counts per section compared to PAS- and AB-stained sections, further supporting the superior sensitivity of GMS (median: 13,026 vs. 7,408 vs. 697, *p* < 0.001 for GMS vs. PAS and GMS vs. AB).

The GMS staining originated from Gomori’s (1946) histochemical method for detecting glycogen and mucin, which Grocott (1955) later optimized into a specialized fungal identification technique. Similarly, the PAS technique was introduced by McManus for visualizing mucin (McManus, 1946). Both techniques depend on oxidizing agents (chromic or periodic acid) to produce binding sites for silver ions or Schiff reagents via aldehyde formation. Chromic acid in GMS converts hydroxyl groups (-OH) to aldehyde groups (-CHO) and then to carboxyl groups (-COOH). Fungal cell walls, rich in polysaccharides, primarily stop at the aldehyde group stage. Non-fungal tissues, which contain fewer polysaccharides, oxidize fully to carboxyl groups. These carboxyl groups cannot bind silver ions, making GMS highly specific for fungi. In contrast, PAS can stain a wide range of tissue components including glycogen, mucins, and collagen fibers, because periodic acid only oxidizes vicinal diols to aldehydes. However, the overlapping chromatic profiles between fungi and other PAS-positive structures create diagnostic challenges, as colorimetric interference complicates morphological discrimination. Our data indicate that GMS exhibits higher sensitivity than PAS, which is primarily attributed to the superior oxidizing capacity of chromic acid. Although chromic acid and periodic acid differ in their reaction endpoints with ethylene glycol hydroxyl groups, the former’s strong oxidizing ability generates a greater number of aldehyde groups. This enhanced oxidation efficiency significantly boosts GMS sensitivity, making it more effective for detection.

AB staining (pH 2.5) uses a copper-phthalocyanine dye with polycationic side chains, which was first used by Steedman to visualize acidic mucins (Steedman, 1950). This dye binds to the polyanions of carbohydrates, forming blue insoluble complexes. AB stain reacts with multiple tissue components, including cryptococcal capsular polysaccharides, acidic mucins, and connective tissue proteoglycans. When used for fungal staining, it can produce interfering noise that complicates diagnosis. Our data showed that the sensitivity of AB staining for *Cryptococcus* detection was only 75.4%. This limited performance is primarily due to two key biological factors: Firstly, some strains of *Cryptococcus* naturally lack complete capsules. Secondly, during the processing of tissue specimens, organic solvents can damage the cryptococcal capsules, causing shrinkage or lysis (El-Zammar and Katzenstein, 2007).

Our observations revealed that *Cryptococcus* organisms exhibit two distinct spatial distributions. IC are completely phagocytosed by macrophages or multinucleated giant cells, while EC remain unphagocytosed, clustered, and surrounded by large, clear halos. Following GMS staining, both IC and EC exhibited strong staining. Fungal elements were distinctly stained in rich brown-black hues, sharply contrasting with the green counterstain. This optimized optical differentiation enhances the visual quality of tissue sections, enabling rapid screening of positive organisms even at low magnification.

Our data also showed that only 5.9% (4/68) of IC and 41.2% (28/68) of EC exhibited strong positivity with PAS staining, while only 2.9% (2/68) of IC and 51.5% (35/68) of EC exhibited strong positivity with AB staining. These strong positivity rates were statistically lower than those of GMS staining (100%, 68/68, *p <* 0.001 for all comparisons). The weaker staining intensities observed in PAS and AB staining are closely associated with the structural disruption of fungal cell walls and capsules. Cryptococcal infections begin with the inhalation of aerosolized spores or desiccated yeast cells. Upon reaching the lungs, fungal cells synthesize and thicken their polysaccharide capsules (Bojarczuk et al., 2016; Maxson et al., 2007). Alveolar macrophages, the primary force of defense in the lungs, recognize cryptococci through surface pattern recognition receptors and phagocytose them (Leopold Wager et al., 2016). Following internalization, reactive oxygen species and reactive nitrogen species produced within macrophages cause oxidative damage to cryptococci (Nelson et al., 2020; Leopold Wager and Wormley, 2015), while lysosomal enzymes further degrade the fungal cells (Mansour et al., 2014). These processes demonstrate the capacity of macrophages to disrupt both the cell walls and capsules of IC. Similar structural damage was observed in cryptococci within multinucleated giant cells, which may be due to damage caused by macrophages prior to fusion or to the direct antimicrobial activity of these giant cells themselves. However, the direct killing ability of multinucleated giant cells remains controversial (Ahmadzadeh et al., 2022), and their specific molecular mechanisms require further exploration in our subsequent studies. Although cryptococci are disrupted in the extracellular environment, intracellular conditions result in more effective killing and pronounced disruption of their polysaccharide structures. This leads to the loss of PAS and AB staining targets and a reduction in staining intensities. Consistently, our data showed significantly lower staining intensities for IC compared to EC in both PAS and AB staining (*p* < 0.001). In contrast, GMS maintains a sharp staining effect due to its high sensitivity.

Necrotic changes were identified in 14 (20.6%) of the 68 PC cases. GMS staining demonstrated strong positivity in both necrotic cores and adjacent peri-necrotic margins. In contrast, PAS and AB staining intensities were significantly higher in the peri-necrotic margins than in the necrotic cores (*p <* 0.05 for both comparisons), with elevated false-negative rates observed in the latter. In regions of tissue necrosis, *Cryptococcus* organisms underwent degradation and subsequent cell death, accompanied by progressive disintegration and eventual disappearance of their capsules and cell walls. This process led to a reduction in fungal polysaccharide content and structural fragmentation of the polysaccharide components. Consequently, these pathological changes manifested as significantly diminished staining intensities or even complete negativity in both PAS and AB. Given these findings, PAS and AB staining should be used with particular caution in necrotic cases, especially when biopsy tissue samples are limited. In contrast, the GMS staining method demonstrated high sensitivity and efficacy in detecting microscopic fungal cell wall remnants, showing notable stability and reliability. Despite this advantage, the cryptococcal density in necrotic cores was lower than that in peri-necrotic margins, likely due to the disappearance of cell walls following extensive organisms’ death in the necrotic cores. This finding suggests that pathologists should prioritize examining peri-necrotic margins when cryptococci are not identified in necrotic cores.

## Conclusion

GMS staining is superior to PAS and AB for diagnosing pulmonary cryptococcosis, as it exhibits higher sensitivity and enhanced visualization of cryptococci, particularly in intracellular compartments, necrotic foci, and colonies with low structural polysaccharide content. GMS is recommended for accurate pathological diagnosis.

## Data Availability

The original contributions presented in the study are included in the article/Supplementary material, further inquiries can be directed to the corresponding author/s.
