# Supplementary material for: Superior diagnostic performance of Grocott methenamine silver staining in pulmonary cryptococcosis: a multicenter, large-sample cohort study
Source: Front Microbiol. 2025 Jun 4;16:1615057. doi: 10.3389/fmicb.2025.1615057 (PMC12174090; doi:10.3389/fmicb.2025.1615057)
Supplement: Supplementary file 1 [file Table_1.DOCX]

**Supplementary Table S1.** Descriptive statistics of cryptococcal counts detected by GMS, PAS, and AB staining

| **Statistic** | **GMS** | **PAS** | **AB** |
| --- | --- | --- | --- |
| n | 68 | 68 | 68 |
| Median | 13026 (34–320600) | 7408 (0–226352) | 697 (0–179371) |
| 25% Percentile | 5316 | 2194 | 34 |
| 75% Percentile | 32303 | 17232 | 6904 |
| Mean ± SD | 31329 ± 56535 | 19905 ± 40908 | 10616 ± 29162 |
| Sum | 2130360 | 1353539 | 721891 |
